# Ten years of *FEBS Open Bio*

**DOI:** 10.1002/2211-5463.13327

**Published:** 2021-11-17

**Authors:** Duncan E. Wright, Miguel A. De la Rosa

**Affiliations:** ^1^ *FEBS Open Bio* Editorial Office Cambridge UK; ^2^ Institute for Chemical Research (IIQ) Scientific Research Centre Isla de la Cartuja (cicCartuja) Universidad de Sevilla‐CSIC Spain

## Abstract

This month, *FEBS Open Bio* celebrates its 10th birthday. To celebrate the journal's first decade, we present this special anniversary issue, comprised of editorials, reviews, and research articles especially commissioned for the occasion. In this introductory editorial, we invite the reader to join us as we reminisce over the journal's past, celebrate its present, and look forward to its future.

## Graphical abstract

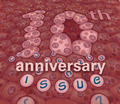

This month, *FEBS Open Bio* celebrates its 10th birthday. From publishing only four articles in its first volume in 2011 to publishing over 260 articles in 2021, the journal has come a very long way. Many factors have contributed to the journal’s success, including the authors who choose to submit their work, the academic editors and reviewers who generously contribute their time and expertise, the readers who download, share and build on the work, and of course, the tireless efforts and commitment of the editorial office staff, the FEBS Publication Committee, the FEBS administrative staff and our publishing partners, Elsevier (2011–2015) and Wiley (2016–). The greatest contributing factors to the journal’s success are the dedication, experience and knowledge of the journal’s original Executive Editor, Mary Purton, who for eight years managed and guided the journal from its very beginnings to a recognised and respected journal and who continues to oversee *FEBS Open Bio* and its companion journals as FEBS Press publisher.

The journal is the youngest of four siblings: *The FEBS Journal* is now 54 years old (or 115, if you include its original incarnation as the German language journal *Biochemische Zeitschrift*), *FEBS Letters* is a bit younger, at 53, and *Molecular Oncology* is now 14. *FEBS Open Bio* was FEBS’ first fully open‐access journal. We are proud to be part of this illustrious publishing heritage and fully subscribe to the FEBS Press motto of ‘science publishing by scientists’: all submissions to *FEBS Open Bio* are considered by editors who are active scientists and are reviewed by researchers working in the field of study.

To celebrate the journal’s first decade, we present this special anniversary issue, comprised of editorials, reviews and research articles especially commissioned for the occasion. This issue has been a year in the making, and we are sure there is something to interest everyone in its virtual pages. But first, please join us as we reminisce over the journal’s past, celebrate its present and look forward to its future.

## A brief history of *FEBS Open Bio*

Following the move to digital publication at the end of the 20th century, the movement towards open‐access publishing began to gain momentum at the start of the 21st century. By 2011, recognising that the move towards open access could not be ignored, the Federation of European Biochemical Societies (FEBS) set up a fully open‐access and online‐only journal. Further details of the journal’s genesis are provided in the following editorial [1].

The first issue of the journal was published in December 2011, with Mary Purton appointed as Executive Editor. Initially, most articles were transferred from other FEBS Press journals, but direct submissions were also received. In those early days, the journal benefitted from the expertise and support of the Editors in Chief of *The FEBS Journal* (Richard Perham), *FEBS Letters* (Felix Wieland) and *Molecular Oncology* (Julio Celis).

In 2016, the journal transferred to Wiley, joining *The FEBS Journal* and *FEBS Letters* to form FEBS Press, allowing the journals to be better consolidated with the other activities of FEBS. *Molecular Oncology* transferred a year later (flipping from a subscription to an open‐access journal at the same time), finally bringing all four journals together under the FEBS Press banner.

In 2017, the journal started a new Education section, for the publication of research on biochemistry education. This section, which is edited by Angel Herráez and Luciane V. Mello, was introduced to encourage research into innovative teaching methods for biochemistry and the dissemination of best practice. The publication costs of all Education articles published in the journal are covered by FEBS, and so, there is no cost to either author or reader. At the time of writing, 16 Education articles have been published in this ‘journal within a journal’, many of which have been well‐read and cited. We are proud to be the venue for many excellent and freely accessible education articles, as effective training of the next generation of biochemists and molecular biologists is essential for the future of research.

As the journal settled in with its new publisher, direct submissions continued to grow and the editorial office support was expanded with the appointment of Kerry Dresser as Editorial Assistant in 2017 and Duncan Wright as Editorial Associate in 2018. Jacob Weller replaced Kerry in 2018. By 2019, it was apparent that the time had come to separate the role of Executive Editor into two distinct positions, an academic Editor‐in‐Chief to set journal strategy and a full‐time Editorial Manager to handle the day‐to‐day operations of the journal. In 2019, the FEBS Publications Committee appointed Miguel A. De la Rosa as the journal’s first Editor‐in‐Chief, with his term commencing on 1^st^ January 2020 [2]. Mary was promoted to FEBS Press Publisher, to work with the Publications Committee and Wiley on FEBS Press strategy, and Duncan was appointed as the journal’s Editorial Manager.

Under Miguel’s Editorship, the journal has continued to grow, not only in terms of number of submissions and acceptances but also in visibility and variety of journal content. Downloads of articles published in the journal continue to increase dramatically year by year: downloads from the journal website alone (not including downloads of the articles from other sources) increased by a third from 2019 to 2020, and by the end of August 2021, downloads were already 6% greater than in the total of 2020, suggesting that articles published in the journal are useful to the community.

Miguel has also implemented several new initiatives to improve journal performance and diversify its content, which are summarised below.

### Expansion of the editorial board

When *FEBS Open Bio* was first founded, its editorial board was comprised entirely of members of the editorial boards of the other FEBS Press journals; many of these original editors are still with the journal, and we are exceedingly grateful for their long service and dedication. As submissions to the journal increased, it soon became apparent that more editors were needed, to handle not only a greater quantity of manuscripts but to also ensure we had the expertise to handle manuscripts on diverse subjects. In addition, we have considered the geographic spread and gender balance of the board in our appointments, to help ensure that our editors best reflect our authors and readers. The editorial board was expanded in 2013 and 2018, and in 2020 and 2021, we appointed fifteen and two new editors, respectively. We are very pleased that all of our new appointments have assimilated well into the journal environment, and we look forward to continuing to work with them for many years.

### Expansion of the editorial office

At the beginning of 2021, Ruzhica Bogeska was appointed as Assistant Editor working across *FEBS Open Bio* and *Molecular Oncology*. Ruzhica has brought many years of postdoctoral experience to the role; she completed her PhD at the University of Freiburg, followed by postdoctoral positions at University Medical Center Freiburg and subsequently the German Cancer Research Center (DKFZ). Ruzhica quickly became an integral part of both the journal teams, spearheading new initiatives and drawing on her experience and network to promote the journals. We are exceptionally grateful to Ruzhica for her initiative, professionalism and attention to detail, which has elevated the journal. We have also recently appointed Ioannis Tsagakis as a second Assistant Editor working across both *FEBS Open Bio* and Molecular Oncology; Ioannis completed a very successful internship at FEBS Press last year, at which time he impressed everyone with his diligence and enthusiasm.

### Editorial advisory board

When it comes to journal performance, our top priority is to maintain thorough and timely peer review of all submissions. To ensure that we always have willing, available and expert reviewers, we introduced a new Editorial Advisory Board (EAB) in 2020. The members of our EAB are committed to providing assistance to our editors, through offering independent evaluations when reviewers disagree, providing thorough peer review at short notice and ensuring that authors have appropriately revised their manuscript when the original reviewer is no longer available to check. At present, the EAB is comprised of 34 members, and we hope this number will increase in future to better reflect the broad scope of the journal.

### Pool of volunteer reviewers

With the journal regularly receiving over a hundred submissions a month, it is important that we have access to a large pool of dedicated and experienced reviewers with diverse research interests. While the EAB is very valuable in this regard, its numbers are too few to review the majority of submissions to the journal. To meet the growing need for reliable reviewers, we reinvigorated our volunteer reviewer pool in 2020 by inviting new members, ensuring that our records on reviewers’ research interests and contact details were current and encouraging our editors to make full use of our volunteers. We continue to publish an annual Reviewer Acknowledgement in December to thank all of our reviewers, and this year is no exception – the Acknowledgement is included at the end of this issue.

### The journal’s first Editorial Board meeting

The journal had its first editorial board meeting in October 2020 – while we had all been looking forward to meeting each other in Seville and enjoying a drink in the warm weather, fate had other plans. Once it had become apparent that there was to be no travel that year, the decision was made to have a virtual meeting and postpone the in‐person meeting to a later date. We did find that the virtual meeting had its boons – it was far better attended than could be expected of a physical meeting, with editors attending from all over the world and not just Europe. We consider this editorial board meeting a major milestone for the journal, and it provided a sense of community, involvement and journal ownership to our editorial board.

### ‘In the Limelight’

The journal has also started to publish special ‘In the Limelight’ issues, which include review articles focused on a topic of especial interest. Our first ‘In the Limelight’ section focussed on the important issue of Bioplastics and was commissioned to accompany the FEBS Special Session on Science & Society – Plastics: revolution, pollution and substitution at the 45th FEBS Congress, which at the time was scheduled for July 2020. We fully expected that the special section would materialise after the Congress, little expecting that the event would be postponed a year and then transformed into a virtual event. As it happened, the Bioplastics section preceded the Science and Society session by four months, and we hope it was able to stimulate interest in the event [3].

The reviews for the ‘Membraneless organelles’ section were also commissioned to tie in with a symposium to be held in Seville in March 2020, which was postponed and then unfortunately cancelled. Despite this setback, the silver lining was that the accompanying issue was published in September of this year. The three review articles in this issue highlight very different factors that are involved in the condensation of membraneless organelles [4]. We are very grateful to the authors who contributed such excellent articles to our first two ‘In the Limelight’ sections and are confident that future special sections will continue this trend of high‐quality reviews.

### Insight articles

To present articles published in the journal to a lay audience, we started a new series of articles called ‘Insights’ in 2021. This series, coordinated by our editor Cornelia de Moor, aims to present findings with broad significance to a general audience. In these Insight articles, the main findings are summarised and presented within a wider context. To date, we have published four Insight articles, two of which were written by postdocs interested in science communication, while the other two were written by editorial office staff. We consider it very important for the journal to be involved in outreach activities and are delighted that this series has had such a productive start in its first year. We invite any researchers interested in contributing to this series to contact the editorial office.

### Interviews with our editors

We are extremely proud of our prestigious Editorial Board. Although they are the central pillars of the journal, their contributions are largely ‘behind the scenes’ and not visible to the reader. We decided that the 10^th^ anniversary of the journal was an ideal time to give our editors a platform to discuss their research and other topics of interest. Our editor Beáta Vertessy agreed to help coordinate this series, dubbed ‘An open chat with…’, and to date, we have published two interviews, with more ready to be published in 2022.

### Closer ties with the annual FEBS Congress

Since 2018, the journal has published the talk and poster abstracts for the annual FEBS Congress as a special supplement issue. Building on this tradition, Miguel has introduced new initiatives to strengthen the ties between the journal and the Congress, as well create ties with the annual Young Scientists’ Forum (YSF), which precedes the Congress. The first of these new developments was the introduction of two *FEBS Open Bio* prizes for the Speed Talks presented at the FEBS Congress. At this year’s 45^th^ FEBS Virtual Congress, a jury comprised of members of the local organising committee and the journal’s editorial office selected two excellent Speed Talks, the presenters of which won €200 each. The winners were:

1. Lígia Fão of University of Coimbra, Portugal, for the speed talk ‘Altered autophagy‐dependent c‐Src/Fyn degradation in Huntington’s disease – impact on NMDAR activity’.
2. Natalia Kruglova of the Institute of Gene Biology RAS, Moscow, Russia, for the speed talk ‘Cell‐to‐cell transmission of SARS‐CoV‐2 is resistant to neutralizing antibodies’.

In addition, the journal continues the tradition of awarding a prize to the presenter of an excellent poster; this year’s FEBS Open Bio Poster Prize was awarded to:

1. Martin Toul of Masaryk University, Brno, Czech Republic, for the poster ‘Engineering protein dynamics for the understanding of divergent evolution of the Renilla luciferase’.

The 20th FEBS YSF, originally planned for Lovran, Croatia, in 2020, and then held as an online event in 2021, brought (virtually) together about 100 PhD students and postdoctoral researchers, to present their research, discuss their experiences, participate in workshops on career skills (including one held by our editorial manager) and attend lectures by distinguished speakers. At this year’s event, the winners of the best short oral presentation and best poster received discounts on the article processing charge for future submissions to the journal. The winners were as follows:

Best short oral presentation (SOP) prize: Angela Garcia‐Mato of the Instituto de Investigaciones Biomédicas ‘Alberto Sols’ (IIBM), Madrid, Spain, for the talk ‘IGF‐1 via PI3K/AKT activation promotes survival and anabolic metabolism in HEI‐OC1 auditory cells’.

Best poster prize: Carmen Escalona‐Noguero of IIMDEA‐Nanoscience, Madrid, Spain, for the poster ‘Nanoparticle‐mediated delivery of Cpf1 for the generation of improved gene editing tools’.

We would like to take this opportunity to congratulate the winners above as well as the winners of the Congress daily poster prizes and wish them all the best with their future careers.

### The *FEBS Open Bio* article prize

This year also saw the introduction of the *FEBS Open Bio* Article Prize, which is awarded to an early‐career researcher who has authored (either as first or corresponding author) an article of especial interest published in the journal in a 12‐month period from July to June of the previous year.

A jury of three members of the journal’s Editorial Board, Stuart Ferguson (Oxford), Takashi Gojobori (Mishima) and Alex Wlodawer (Frederick), selected the following as the winning paper: ‘HSF1 is required for induction of mitochondrial chaperones during the mitochondrial unfolded protein response’, authored by Arpit Katiyar, Mitsuaki Fujimoto, Ke Tan, Ai Kurashima, Pratibha Srivastava, Mariko Okada, Ryosuke Takii and Akira Nakai of Yamaguchi University School of Medicine, Ube, Japan [5]. In this article, the authors showed that the heat‐shock transcription factor HSF1 is required for activation of mitochondrial chaperone genes in mouse embryonic fibroblasts during impaired mitochondrial proteostasis. The *FEBS Open Bio* Article Prize was awarded to the paper’s first author, Arpit Katiyar, who is now a postdoctoral researcher at SGPGI Lucknow, India. Arpit has been invited to submit an abstract on his current work for presentation at the 46th FEBS Congress to be held in Lisbon, Portugal, 9–14 July 2022. In addition, Arpit has also created a video describing the award‐winning work, which can be viewed here: https://febs.onlinelibrary.wiley.com/journal/22115463/prize‐winner

Congratulations to Arpit, and we look forward to seeing you in Lisbon!

## Celebrating the 10^th^ anniversary

The idea for the special issue emerged in October 2020, following discussion of the upcoming 10^th^ anniversary between the journal’s management team and Mary Purton. Miguel suggested that we mark the occasion in some way and Duncan proposed an entire issue dedicated to the 10^th^ anniversary, with review content and interviews. We invited members of our editorial board to submit review articles and invited both editors and the authors of highly cited articles previously published in the journal to submit their latest research for consideration of inclusion in the special issue.

To mark this special occasion, we knew that we had to have a fantastic cover image. It had to somehow represent the 10^th^ anniversary, but also reflect the journal’s strong history of publishing sound science. Eventually, we decided that this history should be represented by the most cited article in the journal’s history: ‘Extracellular vesicle‐mediated transfer of long non‐coding RNA ROR modulates chemosensitivity in human hepatocellular cancer’ [6]. This article has been cited 265 times at the time of writing, speaking to the impact this article has had on its field of research. But how to combine this paper together with the anniversary message into a single image? Duncan proposed a concept in which hepatocellular tumour cells would assume the shapes of the numbers ‘one’ and ‘zero’, which would in turn release extracellular vesicles that would drift towards the viewer and create the phrase ‘anniversary issue’. The very talented Matthew McClements at Blink Studio refined this initial concept to create a truly excellent cover image. In the final version, we can see a layer of hepatocellular tumour cells, with multiple cells forming the digits. At the top of the image, we can see the TGFβ cytokine, represented as yellow triangles, acting on the tumour cells. The tumour cells are releasing extracellular vesicles, shown in pink, with these vesicles containing the long noncoding RNA ROR (shown as thick blue lines).

## In the issue

We are very excited about the final issue line‐up. In addition to the aforementioned editorial on the journal’s history [1], the article features several excellent research and review articles. A very welcome addition to this 10^th^ anniversary issue is an editorial by Pierre Cosson, a long‐standing member of our Editorial Board, on his experiences developing a curriculum for a new Bachelor‐Master course at the University of Geneva [7]. This is a fascinating account of the creation of a curriculum aimed at preparing students for a career in either an academic research laboratory or a private company, which we are sure will be of interest to all educators.

We were delighted at how many members of our Editorial Board agreed to submit review articles for the 10^th^ anniversary. The variety of exciting and cutting‐edge research topics in this issue reflects the diversity of our Editorial Board, and we are sure these articles will be of interest to our readership. Two review articles in this issue focus on neuronal function, from two different angles: biochemistry and phylogeny. In the first article, Sandro Sonnino and colleagues at the University of Milano discuss the beginnings of the elucidation of a ganglioside ‘code’ which may regulate the physiological function of neurons [8]. This review provides fascinating insight into how complex neuronal functions can be derived by taking advantage of biochemical diversity. In the second article related to neuronal function, Takashi Gojobori and colleagues at King Abdullah University of Science and Technology (KAUST) provide a broad overview of both the molecular and physiological bases for memory, as well as our current understanding of its evolution [9]. As physiological data alone are insufficient to elucidate how memory arose, the evolution of genes related to memory must also be studied. Several genes contribute to memory formation, and thus a genomics approach may assist in understanding the evolution of memory.

A fortuitously timed inclusion to this anniversary issue is Maria F. Drincovich’s review of metabolic diversity and reconfiguration in peach fruit [10] – while those of us in the Northern Hemisphere may not associate December with peaches, it is the beginning of the peach season in Argentina, where Maria works at the Universidad Nacional de Rosario. Such examination of the metabolome of peach and other fruits is imperative to ensure food security for a growing global population. We are also delighted to introduce a review article by Laszlo Nagy and colleagues at Johns Hopkins All Children's Hospital, Florida, USA, and the University of Debrecen, Hungary, on transcriptional repressors that modulate macrophage function and development [11]. Research in this field is important not only for facilitating the identification of new therapeutic targets in macrophages but also as a model for elucidating the transcriptional pathways that govern cell identity. This article will thus be of considerable interest to both immunologists and those working on genetic and epigenetic control of cell fate.

Autophagy is integral for cell function, and we are pleased to include two review articles on different aspects of this multifaceted process. Mitophagy is a specialised form of autophagy that primarily removes damaged and dysfunctional mitochondria, but it may also play a role in the development of specialised cell types through an independent mechanism. In the first of our review articles on autophagy, Ivana Novak and colleagues at the University of Split, Croatia, describe the role of the mitophagy receptor BNIP3L/NIX in the development of certain cell types and outline the gaps in our knowledge of the mechanisms involved [12]. This minireview is a fascinating summary of a field that may have implications for the treatment of certain conditions, including cerebral ischaemic injury. A second specialised form of autophagy is xenophagy, which targets intracellular microorganisms; elsewhere in this issue, Gábor Juhász and colleagues at Eötvös Loránd University, Budapest, Hungary, consider this process in terms of the interactions between proteins of different pathogens (including bacteria, intracellular parasites and viruses) and certain autophagy‐related proteins (Atg8‐family proteins) through the LIR motif [13]. In this context, Gábor and colleagues present a case study of the use of prediction software to identify LIR motifs within SARS‐CoV‐2 proteins and Atg9 proteins, providing a timely example of the importance of this work [13].

The above case study highlights the importance of combined computational and experimental methods in the study of pathogens, and this is further emphasised in two excellent minireviews that focus on specific viral and bacterial proteins, respectively [14, 15]. In the first article, Diana Lousa and Cláudio M. Soares of NOVA University Lisbon focus on the influenza fusion process [14]. Influenza virus continues to be a seasonal danger, causing hundreds of thousands of deaths every year. Current therapeutic strategies mainly treat symptoms, and thus, there is a clear need for novel therapeutic interventions that target the virus. One possible means of preventing infection is inactivating fusion of the host and viral membranes by targeting the influenza fusion peptide (IFP). This timely review summarises the findings of experimental and computational studies of IFP’s structure, mode of action and key residues [14]. In the second minireview, Alberto Alape‐Girón and colleagues at Universidad de Costa Rica, San José, Costa Rica and Instituto Tecnológico de Costa Rica, Cartago, Costa Rica, discuss structural aspects of bacterial phospholipase C (PLC) proteins which exhibit both phosphatidylcholinesterase and sphingomyelinase C activities [15]. Structural insights into these proteins provide important information on their modes of action and cytotoxic effects.

We are also very pleased to introduce the four research articles that were commissioned especially for this issue. Tushar Patel, the author of the most highly cited article in the journal’s history (which is also featured on the cover of this issue) [6], reports in this issue that extracellular vesicles within the cellular secretome can promote the growth of cholangiocarcinoma through the transfer of microRNA [16]. These findings suggest novel strategies of suppressing cholangiocarcinoma growth through extracellular vesicle‐based therapeutics. This issue also features a second research article by an author of a highly cited article published previously in the journal (‘Differential heme release from various haemoglobin redox states and the upregulation of cellular heme oxygenase‐1’ [17]): Abdu I. Alayash reports here that the antioxidant caffeic acid has antisickling properties and thus may have therapeutic potential for the treatment of sickle cell disease [18]. We are pleased that these authors’ earlier articles have been so well cited in their respective fields and are grateful that the authors have returned to publish their latest work with us.

We are also delighted to be able to include two exciting research articles from members of our Editorial Board. Dietmar Manstein and his colleague Simon Hennig of Hannover Medical School submitted an article demonstrating that combining quantum dot triexciton imaging (QDTI) with laser scanning microscopy can increase lateral and axial resolution [19]. This method is reported to be easy to perform and does not require special sample preparation or postprocessing, and we are sure it will thus be of great utility in many fields. Finally, Professor Irene Díaz‐Moreno and colleagues at the University of Seville describe in this issue the functional significance of acetylation of lysines 8 and 53 in cytochrome *c* through site‐directed mutagenesis [20]. Mimetic acetylation alters both thermal stability and various functional properties of cytochrome *c*, furthering our understanding of its extramitochondrial roles.

## Special thanks

Finally, we would like to thank everyone involved in the success of the journal. The journal is the sum total of its published articles, its editorial board and all the time and expertise contributed by authors, reviewers, editors, editorial office staff and the staff of our publishing partners. It is our hope that increasing numbers of authors will choose to publish their work in *FEBS Open Bio* in the future because they anticipate a smooth, friendly publishing experience, and want their article to be free to read immediately on publication in a journal that is committed to maintaining the integrity of the publication record.

We would like to extend our gratitude to the following:

1. The former and current FEBS Secretary Generals, Israel Pecht and Vaclav Paces; the former and current FEBS Treasurers, Iain Mowbray, Alan Fersht and Frank Michelangeli; the former and current Publications Committee Chairs, Félix M. Goñi, László Fésüs and Johannes Buchner; and all other Executive and Publications Committee members past and present, for their significant help with the journal’s launch and success.
2. All the members of our Editorial Board past and present, who regularly commit their time and energies to ensure the success of the journal;
3. All the reviewers who kindly gave of their time and expertise to ensure fair and thorough peer review;
4. Everyone who has submitted their work to the journal over the past decade;
5. All former and current members of the FEBS Press Editorial Office, the FEBS administrative staff and all the support staff at both Elsevier and Wiley, for their hard work and encouragement.
6. Very special thanks go to Mary Purton, as without her commitment, experience and many years of service, the journal would not be the success it is today. We hope that we can continue to maintain the very high standards Mary set for the journal, and it is an honour to continue this legacy.

And finally, we thank you, our loyal readers! We hope you enjoy this 10^th^ anniversary issue and will continue to support us during the next decade.
